# In Silico Molecular Docking Analysis of Valproic Acid as an Inhibitor of Bruton’s Tyrosine Kinase: Potential Drug Repurposing for Allergies

**DOI:** 10.7759/cureus.90680

**Published:** 2025-08-21

**Authors:** Ricardo E Martinez-Martinez, Carlos Ivan Calva-Hernandez, Gloria M. Rodriguez-Lopez, Rodolfo Soria-Castro, Bibiana P. Ruiz-Sanchez, Alma D Chavez-Blanco, Sonia M Pérez-Tapia, Claudia G Pavano-Rodriguez, Rommel Chacon-Salinas

**Affiliations:** 1 Immunology, Escuela Nacional de Ciencias Biológicas, Instituto Politécnico Nacional, Mexico City, MEX; 2 Microbiology and Immunology, Facultad de Medicina Veterinaria y Zootecnia, Universidad Nacional Autónoma de México, Mexico City, MEX; 3 Basic Science Division, Instituto Nacional de Cancerología, Mexico City, MEX; 4 Immunology, Centro de Estudios Científicos y Tecnológicos N° 10 (CECyT 10), Instituto Politécnico Nacional, Mexico City, MEX

**Keywords:** btk, in silico docking, molecular coupling, plcγ2, vpa

## Abstract

Mast cells (MCs) participate in inflammation and pathological processes such as type I hypersensitivity. They can recognize type E immunoglobulins (IgE)-coupled allergens through the high-affinity receptors for IgE (FcεRI) membrane receptor, inducing the activation of a signaling pathway in which molecules such as spleen tyrosine kinase (SyK), Bruton's tyrosine kinase (BTK), and phospholipase Cy2 (PLCγ2) participate, promoting MC degranulation by promoting calcium mobilization. Valproic acid (VPA) selectively decreases PLCγ2 phosphorylation without impacting SyK kinase activity. Although BTK acts in parallel to SyK by phosphorylating PLCγ2, it has not been demonstrated whether VPA directly affects BTK. For this reason, the purpose of this work is to evaluate the interaction of BTK with VPA in an in silico molecular coupling analysis. Three-dimensional (3D) models of the ligand and receptor were selected to evaluate coupling with the support of bioinformatics tools, compared with a reference drug. Our results indicate that VPA binds favorably to BTK near its catalytic site. This binding is distinct from irreversible inhibitors, suggesting its role as a reversible inhibitor of this enzyme.

## Introduction

Mast cells (MCs) differentiate from pluripotent progenitor cells in the bone marrow [1] through stimulation with different cytokines such as IL-3, IL-4, IL-9, IL-10, and SCF [2]. MCs mature in tissues according to the stimulus of the microenvironment where they will reside, either in the mucous membranes or the connective tissue [3]. Two main phenotypes are known: those that produce chymase, tryptase, and carboxypeptidase (MCTC) and those that exclusively produce tryptase (MCT) [4].

MCs participate in many physiological and pathological events mediated by the release of the preformed components contained in their granules. Various stimuli can induce the activation of MCs and the release of their granular components, one of the main stimuli being mediated by type E immunoglobulins (IgE) [5]. These cells present high-affinity receptors for IgE (FcεRI), which, in the presence of the antigen bound to IgE, favor the cross-linking of these receptors, starting the activation process [6]. FcεRI lacks an enzymatic portion, so inside the cell, it requires binding to other molecules to transduce the signal. The Lyn molecule phosphorylates the receptor's activation domains (ITAM) and then couples to spleen tyrosine kinase (SyK). The latter molecule has the possibility of phosphorylating others, such as phosphatidyl inositol 3-kinase (PI3K), protein kinase C (PKC), Bruton's tyrosine kinase (BTK), and other molecules, which aim to promote calcium mobilization. Likewise, Lyn can phosphorylate phospholipase C (PLCγ), which will activate PKC, and other enzymes such as mitogen-activated protein kinases (MAPK), that favor the release of calcium into the cytosol. MC degranulation is generated through these signaling pathways, where the key proteins are SyK, BTK, and PLCγ [7]. Within the components of MC granules are vasoactive substances such as histamine, heparin, and some enzymes involved in tissue inflammation [8].

The inflammation generated by the hyperactivation of MCs has harmful effects on the body, the main example being type I hypersensitivity associated with pathological entities such as asthma, allergy, and anaphylactic shock [9]. Although there are drugs that limit some of the steps linked to inflammation, the importance and frequency of these conditions have led to the development of numerous investigations that seek therapeutic alternatives. A strategy currently used is to favor the repurposing of drugs, that is, to use medicines that are already approved for treating other diseases and to evaluate whether the physicochemical mechanisms participate in pathways related to other pathologies, in this case, allergies [10].

Valproic acid (VPA) has already been shown to have effects on various components of the immune response [11]. Previous work demonstrated that preincubation of bone marrow-derived murine MCs with VPA reduced FcεRI-dependent degranulation during antigen-promoted cross-linking. There is also a reduction in the production of the cytokines tumor necrosis factor-alpha (TNF-α), interleukin-6 (IL-6), and IL-13, the expression of FcεRI, and the phosphorylation of PLCγ2, compared to MCs activated without the drug. The decrease in degranulation was associated with the reduction of phosphorylated PLCγ2. However, Syk phosphorylation was unaffected [12].

The analysis by molecular coupling or docking consists of predicting the most favorable energetic binding between a ligand and a biomolecule using algorithms executed by computer programs. Among the docking applications is the identification of potential drugs aimed at therapeutic targets [13-15]. Because VPA has previously been recognized as a promising alternative for the control of hypersensitivity [11], in this work, we evaluated VPA as a therapeutic alternative using docking to explore the participation of this drug in the MC activation pathway.

## Materials and methods

Selection of the crystallized BTK protein

In order to perform the molecular docking analysis between VPA and BTK, it was necessary to identify the most suitable crystallized structure of BTK available in public databases. To accomplish that, the full amino acid sequence of BTK was retrieved from the National Center of Biotechnology Information (NCBI) database (NCBI, US National Library of Medicine; Bethesda, MD, USA). Then, a Basic Local Alignment Search Tool (BLAST) search was performed against the Protein Data Bank (PDB) to identify experimentally resolved BTK structures with high identity. Crystals with a higher than 90% similarity percentage were considered as potential candidates. In addition, structural quality parameters provided in the attached validation reports in PDB were considered.

The most suitable candidates were analyzed through the UniProt database (The UniProt Consortium, Boston, MA, USA) to identify the presence of unresolved or missing amino acid sequences in the candidate crystal structures, which could affect the integrity of the binding site, and therefore accuracy of the docking simulation. Additionally, geometric validation of the crystals was carried out with the MolProbity server (Richardson Lab, Duke University, Durham, NC, USA), considering all-atom contacts and stereochemical parameters. All these criteria were used to consider the crystallized protein to be suitable for the subsequent analysis, ensuring the reliability of the molecular docking.

BTK conditioning

Once the most suitable model was selected, it was processed and refined using the Discovery Studio Visualizer (v21.1.0.20298, Dassault Systèmes, San Diego, CA, USA) software. First, it was curated by removing all nonprotein entities, including crystallographic water molecules, metal ions, and co-crystalized ligands, not relevant to the analysis. Then, having only the protein backbone and side chains, hydrogen atoms were added to the structure, with particular emphasis on polar hydrogens, critical for the analysis of hydrogen bonding and electrostatic interactions for molecular docking and dynamic simulations. The resulting structure was exported in PDB format for use in subsequent analysis.

VPA acquisition and conditioning

**Figure 1:**
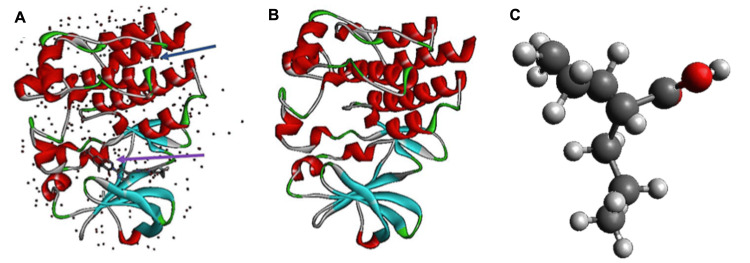
Conditioning and editing of the molecular structure of crystallized BTK (ID: 6XE4) and VPA VPA: valproic acid; BTK: Bruton's tyrosine kinase (A) Three-dimensional structure of the original 6XE4 crystal, purple arrow indicates fluorocyclopropyl amide, blue arrow a sulphate molecule, and dots are water molecules. (B) The edited crystal only shows the protein structure corresponding to BTK. (C) Structure of the edited VPA, each sphere of the model indicates a different atom: red, oxygen; dark grey, carbon; light grey, hydrogen. Images of 6XE4 [16] created with Discovery Studio Visualizer (BIOVIA, Dassault Systèmes (Discovery Studio Visualizer, v.21.1.0,20298), San Diego: Dassault Systèmes, 2021). Image of VPA taken and modified from “Avogadro”: an open-source molecular builder and visualization tool, version 1.2.0, http://avogadro.cc

The two-dimensional (2D) structure of VPA was obtained from the PubChem compound database (NCBI, US National Library of Medicine; Bethesda, MD, USA) of the NCBI, in Spatial Data File (SDF) format. This format preserves atomic coordinates and bonding information for further structural manipulation. This molecule was processed with Avogadro (v1.2.0, Open Chemistry Project, Pittsburgh, PA, USA) software, carrying out an energy minimization employing the Merck Molecular Force Field 94 (MMFF94), a widely used force field for small organic molecules, to obtain the lowest energy conformation of our ligand. Following the minimization, the three-dimensional (3D) structure of this compound was generated and exported in PDB format, as required for most molecular docking platforms (Figure 1).

Search for potential binding sites

To identify potential ligand-binding sites on the BTK protein, a search was performed using DogSiteScorer (ProteinsPlus, Hamburg University, Zentrum für Bioinformatik, Hamburg, Germany). This software uses an algorithm that detects concave regions on the protein surface by analyzing spatial changes in the molecular surface curvature, using a difference of Gaussians, and identifying pockets favorable for ligand accommodation. Each identified pocket is scored based on physicochemical and geometric parameters. Only pockets with a score greater than 0.5 were selected for further analysis, as they are associated with a higher likelihood of representing biologically relevant sites.

Blind docking

Molecular docking simulations were carried out using AutoDock Vina (v1.1.2, The Scripps Research Institute, La Jolla, CA, USA), supported by the MGLTools package (v1.5.6, The Scripps Research Institute, Molecular Graphics Laboratory, La Jolla, CA, USA) and Discovery Studio Visualizer for molecule preparation and visualization. First, MGLTools was used to prepare both VPA and BTK. For the protein, Kollman-type partial charges were added, and for the ligand, Gasteiger-type charges were assigned, following standard docking preparation protocols. Then, both molecules were exported to PDBQT format, as required by the AutoDock Vina software.

A blind docking approach was first explored, where no binding site was specified on the BTK structure. This approach allows the algorithm to explore the whole protein surface for potential binding interactions with VPA. The resulting conformations were ranked based on their Gibbs free energy (ΔG) values in kcal/mol. Those with lower Gibbs free energy values were considered favorable, as they reflect the most thermodynamically favorable interactions. As a positive control, the covalent BTK inhibitor ibrutinib was included in the analysis, as it is widely described that bind directly to cysteine 481 of BTK. This inclusion validates the system.

Targeted docking to potential VPA binding sites and known inhibitors

The above-described docking procedure was subsequently repeated under a targeted docking approach, where specific ligand-binding pockets were defined based on the results obtained from the DogSiteScorer analysis. This strategy allowed a more focused evaluation of potential interactions between VPA and biologically relevant pockets in the BTK protein.

As a positive control, the molecule fluorocyclopropylamide, which is a known BTK-interacting compound, was docked in the highest-scoring pocket. This served to validate the capacity of the pockets to accommodate ligands and provide a reference framework to compare the performance of the analysis with VPA.

## Results

6XE4 was the crystallized protein selected for molecular docking

From the 33 crystals selected by percentage similarity to the amino acid sequence of the BTK protein, homology greater than 90% was sought; only six candidates (Table 1) presented the largest number of favorable parameters, including resolution close to zero and similarity close to 100%. 

**Table 1:** Similarity percentages and quality metrics of candidate BTK crystallized proteins RMDN: Ramachandran; RSRZ: Real Space R value Z-score; BTK: Bruton's tyrosine kinase The identification code of the crystals is determined by the Protein Data Bank for searching within the platform. All selected candidates were free of mutations. The Molprobity rating is a parameter that considers Clashscore, rotamers, and Ramachandran values, normalized on the same resolution scale (X-ray), so a lower value indicates a better rating; the percentile indicates more generally the quality of the crystal, with a value of 100% being the best. Table based on data obtained from Protein Data Bank and Molprobity

Identification code of the candidate crystal	Resolution	Similarity	Free R	Clashscore	RMDN aberrations (%)	Side chain aberrations (%)	RSRZ aberrations	Molprobity score	Molprobity score percentile (%)
	(Å)	(%)					(%)		
5P9F	1.71	99.64	0.224	1	0	0.4	1.1	0.81	100
6XE4	1.6	100	0.18	0	0	0.9	1.1	0.61	100
6AUB	1.65	100	0.199	1	0	0	0.4	0.98	100
6BIK	1.9	99.62	0.222	1	0	0.9	0.4	0.97	100
4OTF	1.95	99.62	0.203	2	0	0	0.4	1.04	100
6AUA	1.66	99.62	0.185	2	0	0.9	1.1	1.08	99

The 6XE4 and 6AUB crystals presented the best parameters. However, 6XE4 was selected because it showed a better free R-value compared to the others. To corroborate this selection, crystal geometry analysis was performed with Molprobity (Table 1), in which 6XE4 presented a better score with respect to the other candidates. In addition, in UniProt, it was observed that the crystal encompasses residues 402 to 655 of the entire protein, which corresponds to the site where the catalytic domain of the protein is located (The UniProt Consortium, 2019), which avoids the need to perform modeling prior to docking analysis. That led to the selection of the BTK 6XE4 crystallized protein as a suitable candidate for molecular docking.

Three potential BTK binding sites were detected

**Figure 2:**
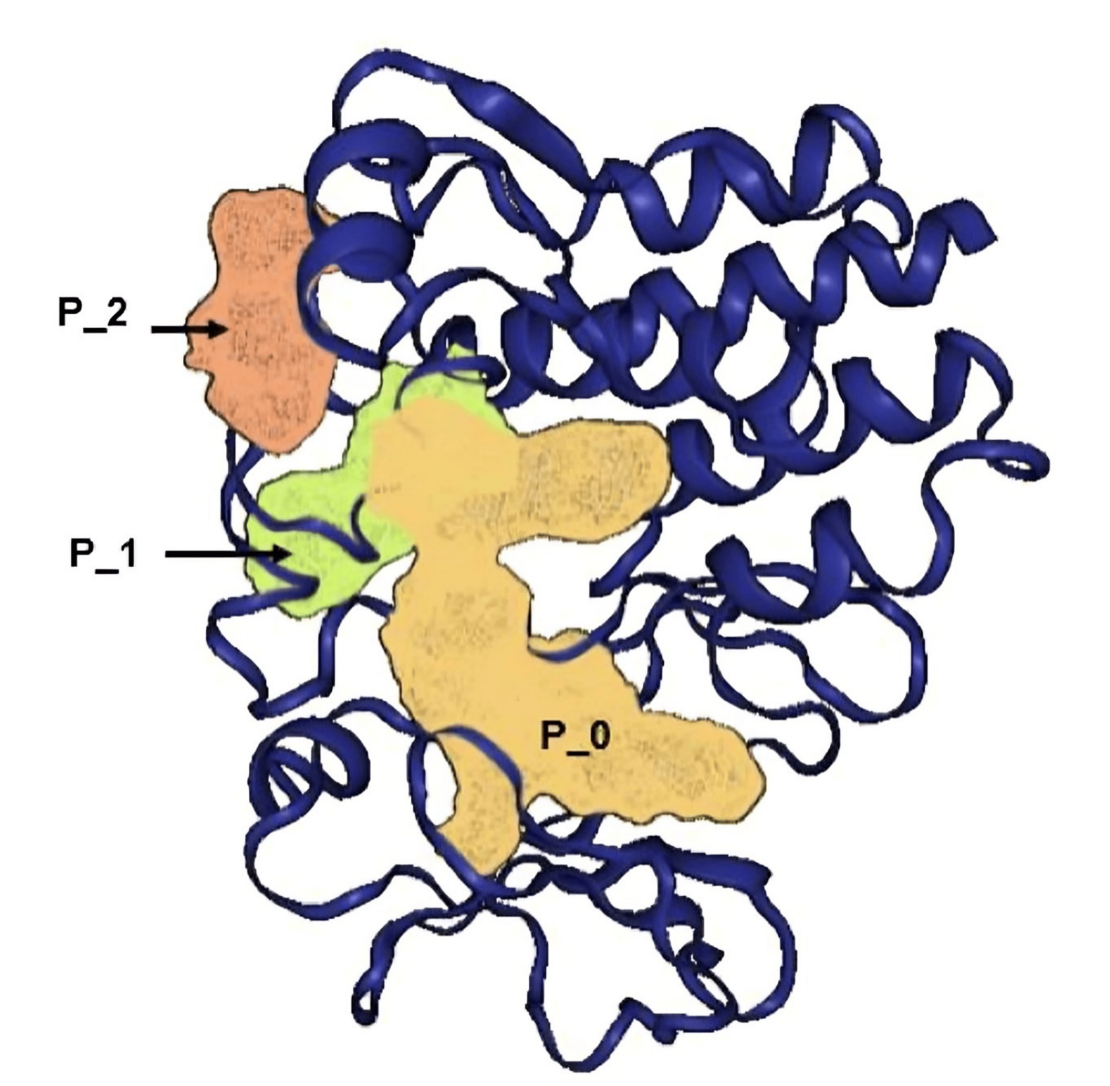
Spatial location of potential binding sites (pockets) in the 6XE4 crystal Colors represent the space spanned by the pockets with the highest druggability; P_0, yellow; P_1, green; P_2, orange. Image of 6XE4 taken and modified from [17], at https://proteins.plus

Using a difference of Gaussians (DoG) algorithm, three pockets were detected within the structure of the crystallized protein, which had druggability values greater than 0.5 (Figure 2), which were identified as P_0, P_1, and P_3, respectively.

P_0 obtained the highest druggability with 0.81, and among its residues is cysteine 481, which is considered the catalytic residue of the protein when binding to adenosine triphosphate (ATP) [18]. Although P_0 has the best characteristics, all three pockets showed close locations in the spatial structure of the model, even at the primary structure level. Therefore, the three pockets were selected for the directed docking study.

Two tentative binding sites were detected in the blind docking

**Figure 3:**
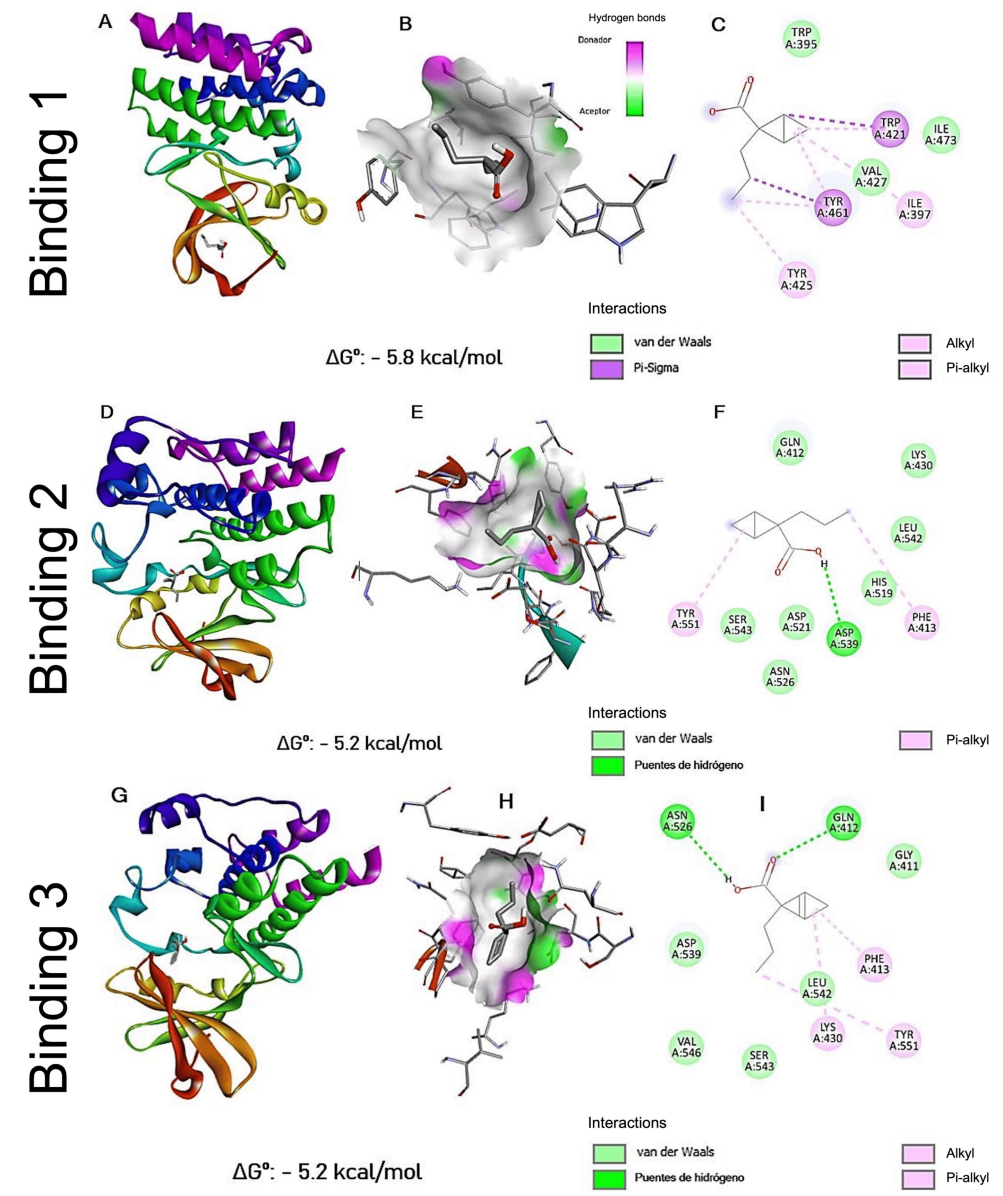
Energetically more favorable interactions resulting from blind docking VPA: valproic acid Panels (A, D, G) show general visualizations of the most favorable interactions between BTK and VPA in three distinct docking conformations using a blind docking approach. Panels (B, E, H) present the molecular surface of the binding site, highlighting the spatial relationship between VPA and the surrounding amino acid residues involved in the interaction. Panels (C, F, I) display two-dimensional interaction diagrams, indicating the specific residues involved and the types of molecular interactions formed (e.g., hydrogen bonds, hydrophobic contacts, electrostatic interactions). For each docking pose, the corresponding Gibbs free energy (ΔGº) value is provided as an estimate of binding affinity. All molecular visualizations were generated using Discovery Studio Visualizer (BIOVIA, Dassault Systèmes)

The results of the blind docking showed two possible binding sites of VPA to the 6XE4 protein (Figure 3); despite having shown a lower binding energy (-5.2 Kcal/mol), one of them was energetically more favorable coupling (-5.8 Kcal/mol), while the other, despite showing a lower binding energy (-5.2 Kcal/mol), was repeated in two of the three most favorable couplings. It should be noted that, in both cases, the free energy obtained evidenced a spontaneous reaction, reflected by Gibbs' free energy [19]. In coupling 1, binding to residues near the catalytic site was observed without showing a direct interaction with cysteine 481, to which the inhibitory drug ibrutinib binds [19]. However, the VPA molecule was coupled near threonine 474, a residue classified as a gatekeeper, located near the entrance of the catalytic site of the protein [20].

Interactions 2 and 3 showed a similar coupling, differing only by some amino acid residues with which they interact, so they were considered as a single coupling. This coupling revealed a noncovalent interaction with tyrosine 551, a critical residue for the transphosphorylation of BTK and directly related to this kinase's activation. 

The results of targeted docking coincide with the more energetically favorable coupling of blind docking

**Figure 4:**
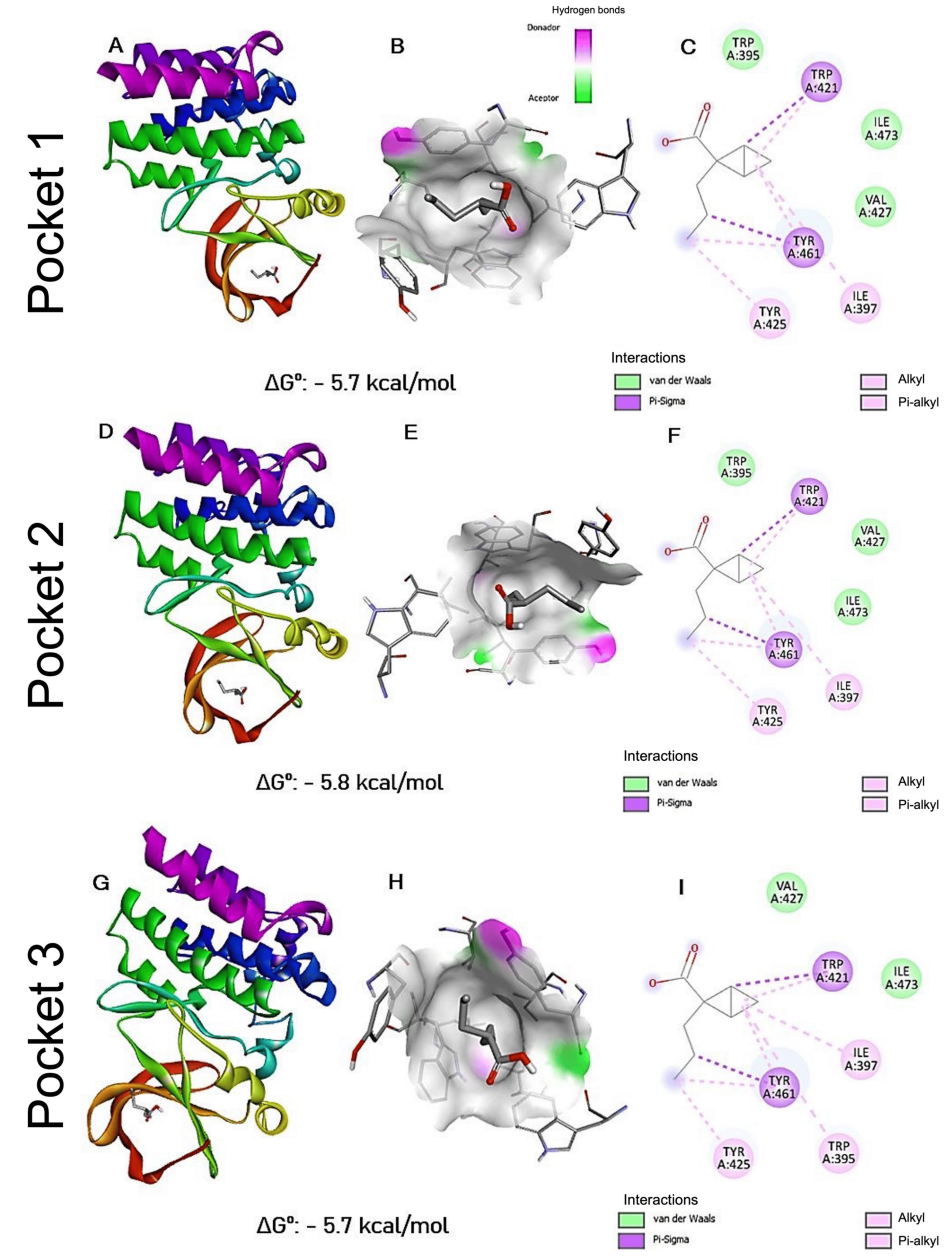
Energetically most favorable interactions directed toward the identified pockets BTK: Bruton's tyrosine kinase; VPA: valproic acid Panels (A, D, G) show general visualizations of the most favorable interactions between BTK and VPA in three distinct docking conformations using a targeted docking approach. Panels (B, E, H) present the molecular surface of the binding site, highlighting the spatial relationship between VPA and the surrounding amino acid residues involved in the interaction. Panels (C, F, I) display the two-dimensional interaction diagrams, indicating the specific residues involved and the types of molecular interactions formed (e.g., hydrogen bonds, hydrophobic contacts, electrostatic interactions). (F) For each docking pose, the corresponding Gibbs free energy (ΔGº) value is provided as an estimate of binding affinity. All molecular visualizations were generated using Discovery Studio Visualizer (BIOVIA, Dassault Systèmes)

A targeted docking was performed using the previously identified pockets. Only the most likely interactions were considered for each of the three cases, and their respective images were displayed the same way as for blind docking (Figure 4). A pattern was found in each of the most favorable joints. A conserved binding pattern was observed across the most favorable docking poses. In all three configurations, VPA interacted with almost the same set of amino acid residues in the binding site: Trp 395, Ile 397, Trp 421, Tyr 425, Val 425, Tyr 461, and Ile 473. The variations observed among the poses were mainly due to differences in the spatial orientation of VPA and minor atomic-level interactions. These subtle variations may account for the small differences in Gibbs free energy observed (ranging from -5.8 to -5.7 kcal/mol). The recurrence of these residues suggests a conserved interaction hotspot within BTK that may be critical for VPA binding.

**Figure 5:**
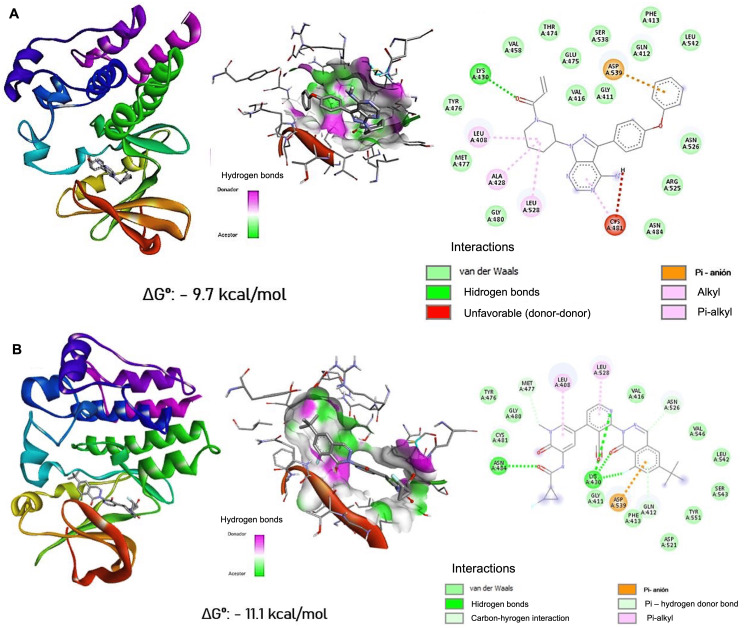
Energetically most favorable binding of ibrutinib and fluorocyclopropylamide (FCPA) to BTK BTK: Bruton's tyrosine kinase Panel (A) general visualizations of the most favorable interaction between BTK and ibrutinib using a blind docking approach, displaying the molecular surface of the binding site and the two-dimensional interaction diagrams. Panel (B) general visualizations of the most favorable interaction between BTK and fluorocyclopropylamide using a targeted docking approach to pocket P_0, displaying the molecular surface of the binding site and the two-dimensional interaction diagrams. All molecular visualizations were generated using Discovery Studio Visualizer (BIOVIA, Dassault Systèmes)

When comparing the binding free energy obtained with VPA with respect to known BTK inhibitors (Figure 5), it was observed that in both cases, the free energy obtained was considerably lower than that obtained by VPA. Notably, ibrutinib showed 21 different atomic interactions. Catalytic residue Cys 481 exhibited an unfavorable donor-donor interaction, as two hydrogen bond donors are oriented too close toward each other without an appropriate acceptor. This type of interaction creates electrostatic repulsion and steric impediment, reducing the overall binding stability. Regarding FCPA, the lowest free energy was observed, highlighting the weak interaction (Van der Waals) with cysteine 481. 

## Discussion

In silico analyses allow us to observe the expected structural behavior of molecules with potential therapeutic use. Currently, one of the most commonly used methods is molecular docking, which allows for structural analysis and potential binding ligand interactions [21]. The determination of molecular structure using crystallography models is a tool that evaluates the formation of complexes between the ligand and the target proteins. In this article, we analyze 33 crystals from an amino acid sequence of the BTK protein, of which six were aligned to the original sequence. However, the crystallized protein 6XE4 proved to be the best option for performing the analysis of molecular coupling, as it met the quality parameters required to be considered a biological representative. This crystal encompasses residues 402 to 655, which include the catalytic domain of the enzyme. Notably, it contains amino acids of functional relevance, such as cysteine 481 (Cys 481), which lies within the ATP-binding pocket and is crucial for its activity. Also, Cys 481 is the covalent binding target of the BTK inhibitor ibrutinib, an irreversible inhibitor used in the treatment of B-cell neoplasms. The presence of this residue in the selected crystal ensures that the docking simulations reflect biologically relevant interactions [22].

On the other hand, the threonine at position 474 (Thr 474) is near the entrance of the catalytic site of the protein [20]. It is considered a gatekeeper responsible for regulating phosphorylation activity and the entry of ERK kinase into the nucleus. Tyrosine 551 (Tyr 551) is involved in the activation of BTK kinase [23] by being identified as a transphosphorylation step that favors the autophosphorylation of BTK in response to Lyn [24]. 

Once the three potential pockets of binding were identified, it was modeled through blind docking analysis using the AutoDock Vina software, which allows the identification of the affinity between the molecules and the favorable energy for the binding between the VPA and the BTK proteins to be accomplished [25]. Three possible junctions were found, one of them with the highest binding strength close to the catalytic site, without coupling to Cys 481. The VPA molecule was coupled near Thr 474. That could hypothetically affect the enzyme's activity by preventing phosphorylation of the catalytic residue of this protein.

The other two junctions behave similarly, with variations in amino acids, so they are believed to form a single junction located in Tyr 551. However, having a higher free energy (-5.2 Kcal/mol), the binding probability is lower. Assuming that in an actual biological model, BTK interacts with multiple VPA molecules, it can be deduced that two different VPA molecules interact with the structure of the enzyme, a first coupling occurring at the most energetically favorable site, followed by a second coupling to the second site found (where the interaction with Tyr 551 occurs), affecting the kinase activity of the enzyme. However, having no tools to demonstrate this phenomenon, the latter is considered speculative.

The targeted docking analysis showed VPA binding in the same region, with the same coupling more energetically favorable for the three cases. That is, despite searching in specific sites of the structure, considering that everything contained within this selection is analyzed by the program's algorithm in the configuration of the search site. The three identified pockets are spatially close, so they had areas of convergence in their respective search sites during coupling. Therefore, it is feasible that said binding site is located in these convergence sites. Since this most energetically favorable binding site in the three pockets analyzed was the same as that obtained during blind docking, it is corroborated that it is the most energetically favorable for VPA. 

On the other hand, two known BTK inhibitors were shown to have lower free energy relative to VPA. The size of the molecules is possibly influenced since it is likely that the greater the number of atoms, the greater the number of atomic interactions. Ibrutinib showed three times more atomic interactions than VPA. Considering that the catalytic residue (Cys 481) showed an unfavorable donor-donor interaction, it is observed that there is a contrast with that reported in the literature since ibrutinib is considered an irreversible inhibitor by forming a covalent bond with said residue [26]. That may be due to the conditions used for docking or to the capacity of the platform used; however, investigating the possible cause of this result is not part of the objective of this work.

On the other hand, FCPA apparently establishes a weak binding (Van der Waals) with Cys 481, demonstrating that the fenebrutinib-derived molecule [15] is a reversible inhibitor since it does not present a covalent bond with the BTK catalytic residue [27].

According to the couplings made in this work, binding with the VPA ligand had the highest free energy compared to the two inhibitors tested, considering that the size of the molecule could influence these data. VPA (144 g/mol) is considerably lower with respect to ibrutinib (440.5 g/mol) and FCPA (510 g/mol); in addition, the atomic interactions between BTK and VPA are considered weak [28]. Despite this, the negative free energy value denoted a spontaneous reaction [19], demonstrating an energetically favorable binding. Such binding was corroborated by repeating the same result under all tested conditions.

The BTK-VPA coupling was presented by interactions with residues near the catalytic site; however, unlike the model drug, VPA did not have direct contact with Cys 481, so under no condition can it be considered an irreversible BTK inhibitor [29]. Despite this, the possibility that it is a reversible inhibitor cannot be ruled out since this type of inhibitor does not precisely require an interaction with Cys 481 to attenuate the kinase activity of BTK [30]. This proposal can be supported by comparing the results obtained with the reversible inhibitors reported in the literature, where these molecules have multiple interactions with various residues of the catalytic domain: fenebrutinib has interactions with residues 430, 477, and 539, while the ARQ molecule shows binding to residues 475 and 476 [31].

Despite the results obtained, this study presents several limitations inherent to the in silico approach used. First, the molecular docking analysis uses a rigid body approach on the protein structure, which does not reflect the flexible nature of proteins under physiological conditions. Also, we used an experimentally resolved crystal, which does not have the complete structure. This may not have an effect directly on the binding sites, but interactions involving other regions cannot be ruled out. Moreover, assessing binding affinity only with Gibbs free energy does not consider all thermodynamic contributions present in a biological environment. All these limitations can be resolved using a dynamic simulation, which can confirm the observed results in a more physiologically relevant system. Finally, our hypothesis of a potential reversible inhibition must be confirmed with complementary in vitro and in vivo experiments.

## Conclusions

This work demonstrated an energetically favorable interaction between VPA and the catalytic domain of BTK. It is proposed that VPA acts similarly to reversible inhibitors, affecting the kinase activity of the enzyme and, consequently, the phosphorylation of PLCγ2. This relationship is consistent with previous research findings, where preincubation of mouse bone marrow-derived mast cells with VPA (prior to antigen challenge) reduced FcεRI-dependent degranulation, in addition to lowering PLCγ2 phosphorylation, without affecting Syk phosphorylation. It should be noted that, due to the restrictions of bioinformatic analyses, it is necessary to conduct experiments to test the hypotheses presented here.
